# First record of *Achalinus zugorum* Miller, Davis, Luong, Do, Pham, Ziegler, Lee, De Queiroz, Reynolds & Nguyen, 2020 from China (Serpentes, Xenodermidae), with updated diagnosis of this species

**DOI:** 10.3897/BDJ.13.e161176

**Published:** 2025-08-22

**Authors:** Shuo Liu, JiShan Wang, Mian Hou, Mo Wang, Dingqi Rao

**Affiliations:** 1 Kunming Natural History Museum of Zoology, Kunming Institute of Zoology, Chinese Academy of Sciences, Kunming, China Kunming Natural History Museum of Zoology, Kunming Institute of Zoology, Chinese Academy of Sciences Kunming China; 2 Yunnan Key Laboratory of Biodiversity Information, Kunming Institute of Zoology, Chinese Academy of Sciences, Kunming, China Yunnan Key Laboratory of Biodiversity Information, Kunming Institute of Zoology, Chinese Academy of Sciences Kunming China; 3 Southwest Survey and Planning Institute of National Forestry and Grassland Administration, Kunming, China Southwest Survey and Planning Institute of National Forestry and Grassland Administration Kunming China; 4 College of Continuing (Online) Education, Sichuan Normal University, Chengdu, China College of Continuing (Online) Education, Sichuan Normal University Chengdu China; 5 Key Laboratory of Biodiversity Conservation in Southwest China (State Forestry and Grassland Administration) / Yunnan Academy of Biodiversity, Southwest Forestry University, Kunming, China Key Laboratory of Biodiversity Conservation in Southwest China (State Forestry and Grassland Administration) / Yunnan Academy of Biodiversity, Southwest Forestry University Kunming China; 6 Kunming Institute of Zoology, Chinese Academy of Sciences, Kunming, China Kunming Institute of Zoology, Chinese Academy of Sciences Kunming China

**Keywords:** COI, distribution, morphology, odd-scaled snake, Wenshan Prefecture

## Abstract

**Background:**

The enigmatic odd-scaled snake *Achalinus zugorum* Miller, Davis, Luong, Do, Pham, Ziegler, Lee, De Queiroz, Reynolds & Nguyen, 2020, was described, based on only one male specimen from Ha Giang Province, northern Vietnam. To date, no other specimen was reported, except for the holotype and this species was considered to be endemic to Vietnam.

**New information:**

Based on one specimen collected from Malipo County, Wenshan Prefecture, Yunnan Province, China, we provide the first record of *Achalinus zugorum* from China. The specimen from China agrees well with the original morphological description of the species and has a minor genetic distance of 2.9% in the COI gene with the type specimen of this species. This study reports the second specimen and the first female specimen of this species. We also provide an updated diagnosis of this species combined with the morphological data of the newly-collected specimen.

## Introduction

The genus *Achalinus* Peters, 1869 belongs to the family Xenodermidae, which is a basal lineage of Caenophidia (Pyron et al. 2013, Zaher et al. 2019, Burbrink et al. 2000). *Achalinus* is a very special group of snakes with a unique non-imbricate scale organisation of dorsal scales that form a dissociated and non-overlapping pattern (Zhao et al. 1998, Zhao 2006, Miller et al. 2020). Currently, this genus contains 29 recognised species, which are mainly distributed in eastern and south-eastern Asia, including Japan, China and Vietnam (Uetz et al. 2025).

*Achalinus zugorum* Miller, Davis, Luong, Do, Pham, Ziegler, Lee, De Queiroz, Reynolds & Nguyen, 2020 is a poorly-known enigmatic species, which was described from Ha Giang Province, northern Vietnam, based on a single male specimen (Miller et al. 2020) and, apart from the holotype, no other specimens of this species have been reported so far. Therefore, this species has been considered endemic to Vietnam.

During our recent herpetological survey in Yunnan Province, China, one female of *Achalinus* was collected. The analysis of morphological and molecular data showed that this specimen belongs to *Achalinus zugorum*. Herein, we record this species from outside Vietnam and from China for the first time. In addition, we provide the first morphological description of the female of *A. zugorum* and provide an updated diagnosis for this species.

## Materials and methods

The snake was collected by hand. After being fixed, it was stored in 75% ethanol. The preserved specimen was deposited at Kunming Natural History Museum of Zoology, Kunming Institute of Zoology, Chinese Academy of Sciences (KIZ).

The methodology of measurements and scale counts followed Miller et al. (2020). The following morphological characteristics were noted: SVL, snout–vent length; TailL, tail length; TotalL, total length; HeadL, head length, from the posterior margin of the parietal scale to the tip of the rostral scale; HeadW, head width, between the widest point of the head; SnL, snout length, from the anterior point of the eye to the tip of the rostral scale; SnW, snout width, distance between the median of the nostrils; EyeD, eye diameter, distance between the anterior and posterior margins of the eye; NarEye, naris–eye distance, from the anterior point of the eye to the medial point of the nostril; InterorbD, interorbital distance, distance between the eyes at the border of the supraoculars; InternasalSL, internasal suture length, length of the suture between the internasal scales; PrefrontalSL, prefrontal suture length, length of the suture between the prefrontal scales; FrontalL, frontal length, maximum length of the frontal scale; FrontalW, frontal width, maximum width of the frontal scale; ParietalSL, parietal suture length, length of the suture between the parietals; LorealL, loreal length, maximum horizontal length of the loreal scale; NasalAH, anterior nasal height, maximum vertical length of the anterior portion of the nasal scale; NasalPH, posterior nasal height, maximum vertical length of the posterior portion of the nasal scale; SL, supralabials; IL, infralabials; ATem, anterior temporals; PTem, posterior temporals; DS, dorsal scale rows, at one head length behind the head, at mid-body and at one head length anterior to the cloacal plate; VS, ventral scales; SC, subcaudal scales; and Prec, precloacal plate. Counts for head scales are given in left/right order.

Total genomic DNA was extracted from liver tissue. A fragment of the mitochondrial cytochrome c oxidase subunit 1 gene (COI) was amplified via the polymerase chain reaction (PCR) using the primers Chmf4 (5′-TYTCWACWAAYCAYAAAGAYATCGG-3′) and Chmr4 (5′-ACYTCRGGRTGRCCRAARAATCA-3′) (Che et al. 2012). Amplification and sequenc­ing were completed by Sangon Biotech (Shanghai) Co., Ltd. Sequences were stitched using SeqMan in Lasergene 7.1 (Burland 2000). Newly-generated sequences have been deposited in GenBank and homologous sequences were obtained from GenBank (Table 1). *Fimbrios klossi* Smith, 1921, *Parafimbrios lao* Teynié, David, Lottier, Le, Vidal & Nguyen, 2015 and *Xenodermus javanicus* Reinhardt, 1836 were used as outgroups according to Huang et al. (2024) and Li et al. (2024).

Sequences were aligned using ClustalW (Thompson et al. 2002) with default parameters in MEGA 12.0.11 (Kumar et al. 2024). Uncorrected pairwise distances were calculated in MEGA 12.0.11. The best substitution model was selected using the Bayesian Information Criterion in ModelFinder (Kalyaanamoorthy et al. 2017). Bayesian Inference was performed in MrBayes v.3.2.7 (Ronquist et al. 2012) using the GTR+F+I+G4 model. The Markov chains were run for 5,000,000 generations with sampling every 100 generations. Maximum Likelihood analysis was conducted in IQ-TREE 1.6.12 (Nguyen et al. 2015) using the TIM+F+I+G4 model with 1,000 ultrafast bootstrap replicates.

## Taxon treatments

### Achalinus zugorum

Miller, Davis, Luong, Do, Pham, Ziegler, Lee, De Queiroz, Reynolds & Nguyen, 2020

54C42782-2EBA-5391-AF9F-DA10AB803B4C

#### Materials

- **Type status:** Other material. **Occurrence:** catalogNumber: KIZ2025021; individualCount: 1; lifeStage: female; occurrenceID: 6FDFDF55-9CDF-58F2-9D9F-20777DE37831; **Taxon:** scientificName: *Achalinus zugorum*; **Location:** country: China; stateProvince: Yunnan; locality: Yunling Village, Xiajinchang Township, Malipo County, Wenshan Prefecture; verbatimElevation: 1450 m; verbatimCoordinates: 23°9'53" N 104°50'44" E; **Event:** eventRemarks: collected on 12 April 2025 by locals; **Record Level:** basisOfRecord: preserved specime

#### Description of the specimen from China

Body size small, cylindrical (SVL 268 mm, TotalL 331 mm), tail moderately long (TailL/TotalL 0.19); head small, elongated (HeadL/SVL 0.03, HeadW/HeadL 0.58), slightly distinct from neck; snout long (SnL/HeadL 0.37), narrow (SnW/HeadW 0.43), tip obtuse; eye quite small (EyeD/HeadL 0.12), pupils vertically elliptical; nostrils orientated laterally.

**Figure 1.**
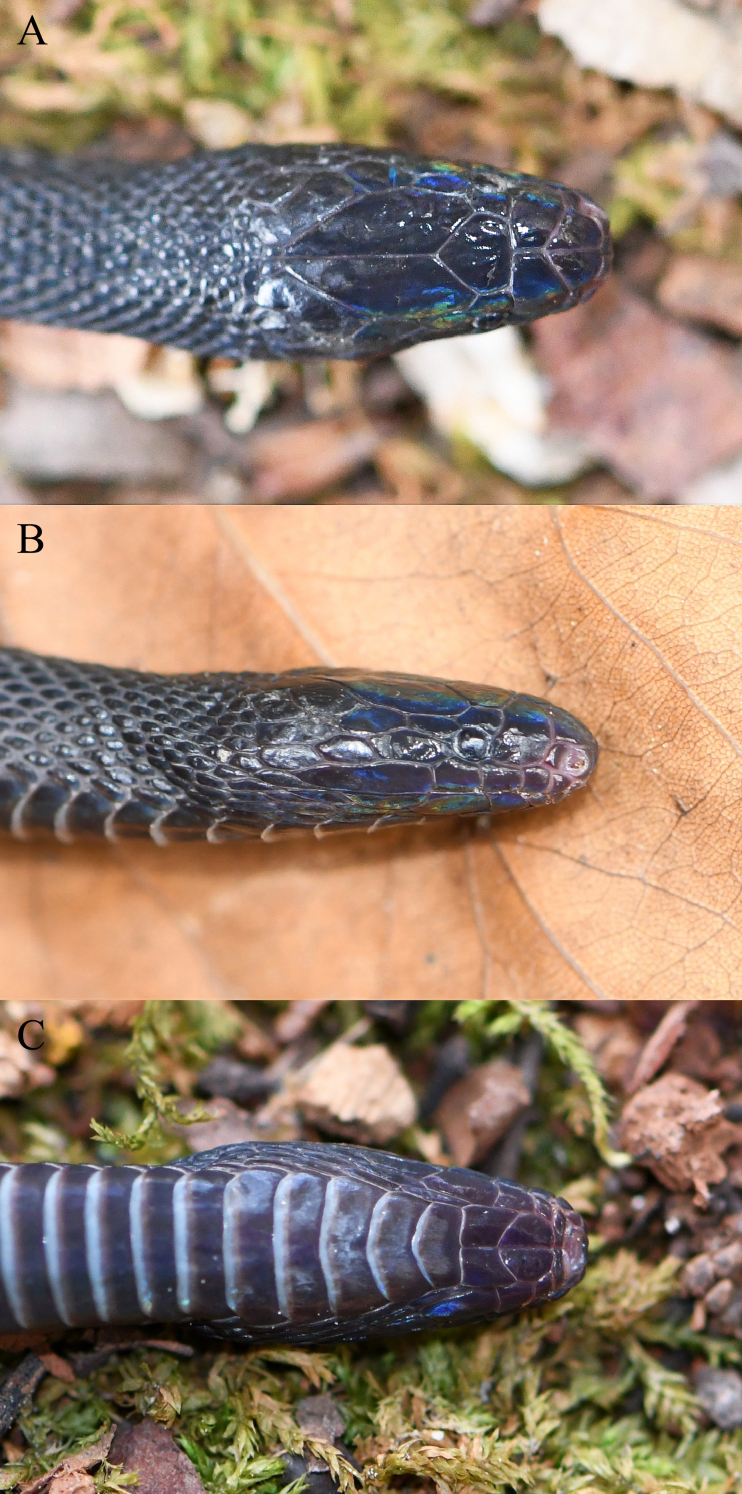
Close-up views of the head of *Achalinus zugorum* (KIZ2025021) from China in life. **A** Dorsal view; **B** lateral view; **C** ventral view.

Rostral small, approximately triangular, slightly invisible from above; nasal divided into two halves; internasals two, prefrontals two, internasal suture longer than prefrontal suture (InternasalSL/PrefrontalSL 2.13); frontal pentagonal, short (FrontalL/FrontalW 0.85); supraocular one on each side, short; parietals two, large, elongate, parietal suture almost two times of frontal (ParietalSL/FrontalL 1.95); loreal fused with prefrontal, in contact with 3^rd^–4^th^ supralabials on each side; preocular and postocular absent; supralabials six on each side, 4^th^–5^th^ entering orbit on each side, last one largest; anterior temporal two on each side, entering orbit; posterior temporals two on each side; mental small, horizontally elongate; infralabials six on each side, first pair in contact with each other after mental, 1^st^–3^rd^ in contact with anterior chin shield; chin shield two pairs; mental groove absent (Fig. 1).

Dorsal scales in 25-23-23 rows, all strongly keeled, diamond-shaped, except for outermost rows distinctly enlarged into approximately triangular shape; ventrals 170, including five preventrals; subcaudals 61, unpaired; precloacal plate undivided.

#### Colouration in life

**Figure 2.**
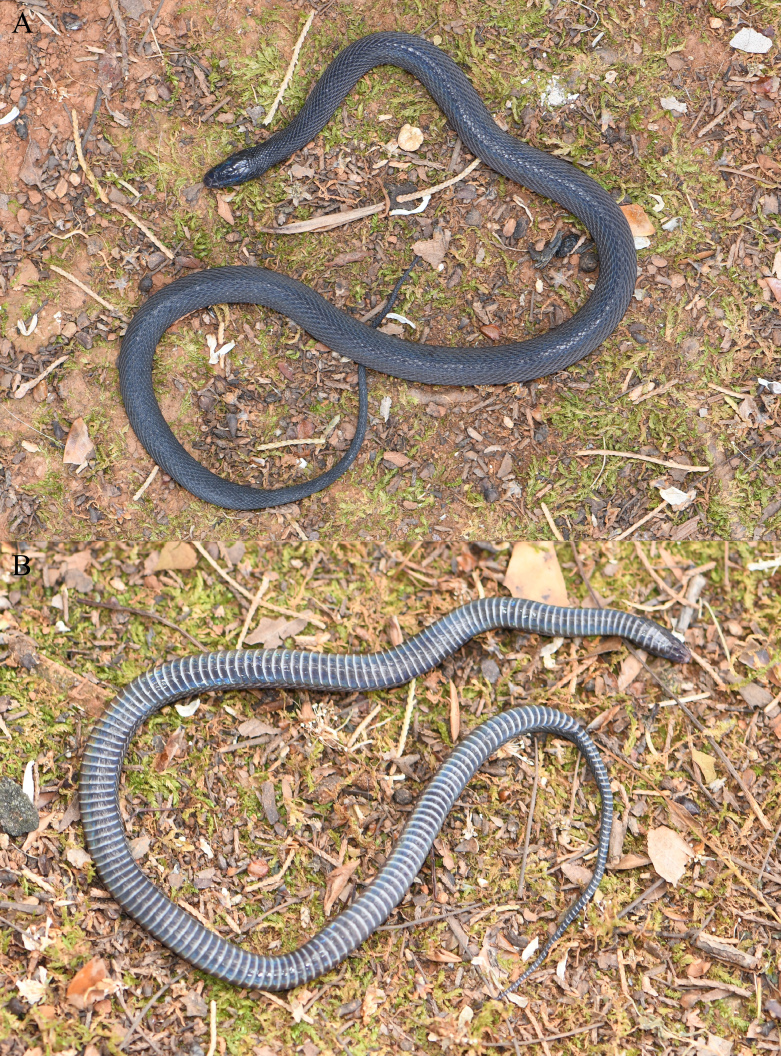
*Achalinus zugorum* (KIZ2025021) from China in life. **A** dorsal view; **B** ventral view.

Dorsal surface of head black, dorsal surface of body and tail greyish-black, chin region purplish-black, ventral surface of body and tail greyish-black with posterior margin of each ventral scale greyish-white, strong iridescence present (Fig. 2).

#### Updated diagnosis

Body size small, total length 331–458 mm; tail length to total length ratio 0.19–0.23; dorsal scales in 25-23-23 rows, all strongly keeled, outermost rows distinctly enlarged; ventrals 170–173, including preventrals; subcaudals 61–70, unpaired; preocular and postocular absent; loreal fused with prefrontal; internasal suture distinctly longer than prefrontal suture; 6–7 supralabials, 6–7 infralabials; mental very thin, separated from anterior chin shields; two anterior temporals entering orbit, two posterior temporals; maxillary teeth 28, all curved and equal in size; dorsal surface greyish-black to jet black, ventral surface greyish-black with posterior margin of each ventral scale greyish-white, scales iridescent in light.

#### Distribution

**Figure 3.**
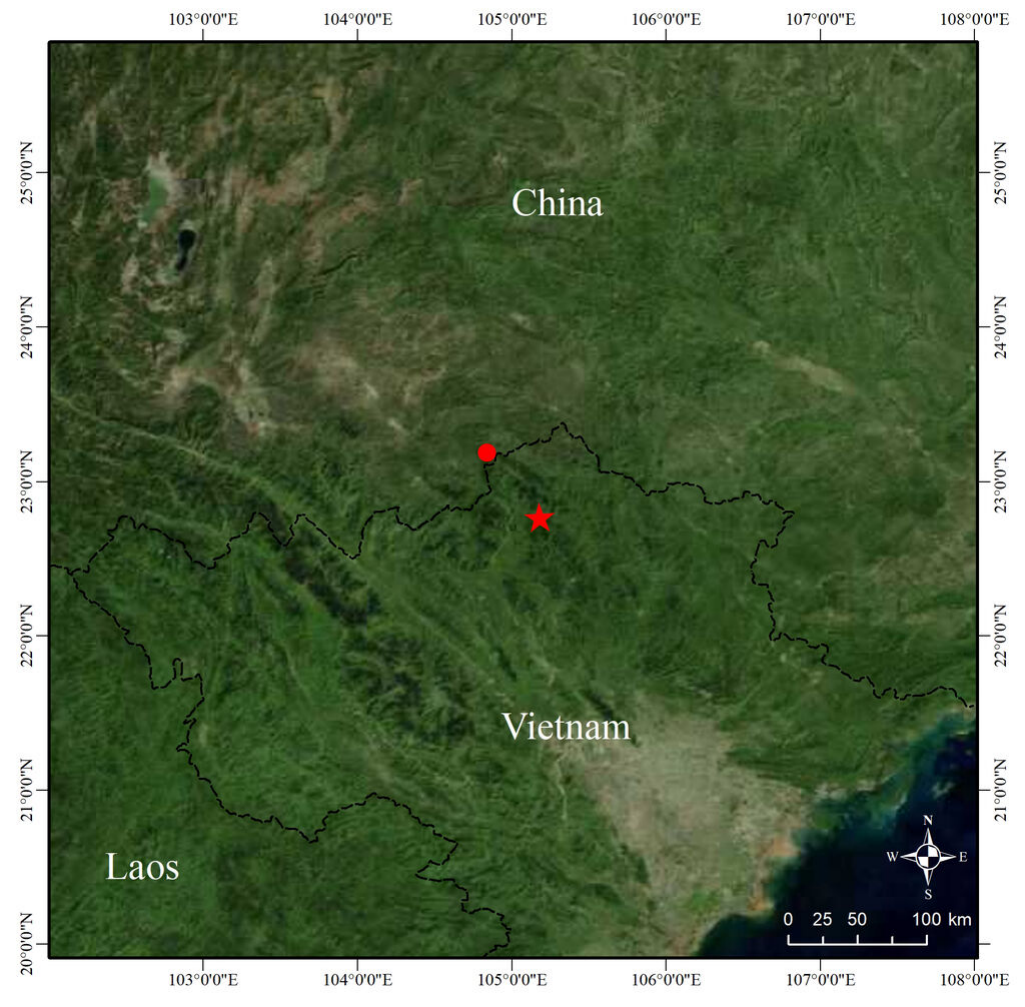
Map showing the type locality of *Achalinus zugorum* in Bac Me District, Ha Giang Province, Vietnam (red star) and the new collection site of the specimen from Xiajinchang Township, Malipo County, Wenshan Prefecture, Yunnan Province, China (red dot).

This species is currently known to be only distributed in Bac Me District, Ha Giang Province, northern Vietnam and Malipo County, Wenshan Prefecture, south-eastern Yunnan Province, China (Fig. 3).

#### Suggested Chinese common name

According to the type locality Ha Giang Province, Vietnam, we recommend 河江脊蛇 (Pinyin: hé jiāng jǐ shé) as the Chinese common name of this species.

## Analysis

Measurements and pholidosis data of the specimen from China are presented in Table 2. The specimen from China agrees with the holotype of *Achalinus zugorum* in most morphological characteristics, except for its having a smaller body size (TotalL 331 mm vs. 458 mm), a relatively shorter tail (TailL/TotalL 0.19 vs. 0.23) and slightly fewer ventral and subcaudal scales (VS 170 vs. 173, SC 61 vs. 70). In addition, there is a small scale between the posterior pair of chin shields and the anterior preventral, while there is no such scale in the specimen from China.

**Figure 4.**
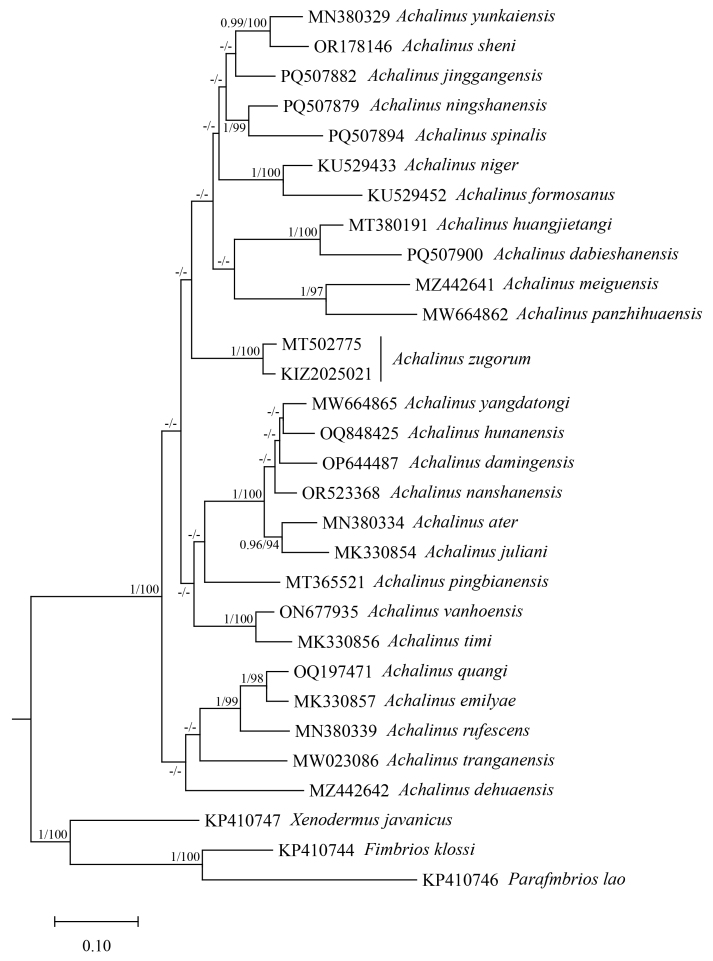
Bayesian phylogenetic tree, based on COI sequences. Numbers after and behind the “/” are Bayesian posterior probabilities and Maximum Likelihood ultrafast bootstrap values, respectively. “-” represents values less than 0.90 or 90.

Molecularly, Bayesian Inference and Maximum Likelihood analysis resulted in similar topologies; therefore, only the Bayesian tree is shown with the Bayesian posterior probabilities and Maximum Likelihood bootstrap values labelled along the branches (Fig. 4). The sequence of the specimen from China clustered with the sequence of the holotype of *Achalinus zugorum* from Vietnam with strong support. The genetic distance (uncorrected p-distance) between the sequence of the specimen from China and the sequence of the holotype of *A. zugorum* was 2.9% (Table 3).

## Discussion

The morphological characteristics of the newly-collected specimen of *Achalinus zugorum* are consistent with that of the holotype of this species, except for some small variations, such as a smaller body size, a relatively shorter tail and fewer ventral and subcaudal scales. The newly-collected specimen is different in sex from the holotype; however, we are currently unable to determine whether these differences are due to sexual dimorphism in this species, since there is only one specimen for each sex. In addition, the difference in posterior chin shields between the holotype and the specimen from China is likely caused by individual variations rather than sexual dimorphism. More specimens are needed to verify whether there are some stable morphological differences between the sexes of this species.

The straight-line distance between the new collection site in China and the type locality of this species is approximately 61 km. The altitude of the type locality of this species is 228 m above sea level (Miller et al. 2020), while the altitude of the new collection site in China is approximately 1,200 m higher than that. Therefore, this species is currently known to inhabit the area between 220 m and 1,450 m above sea level from Ha Giang Province of northern Vietnam to Wenshan Prefecture of southern Yunnan Province, China. Until more data about distribution and population status are available, we do not suggest the threatened assessment of the new species.

*Achalinus zugorum* is a very enigmatic species; since it was described, no specimen has been found until this study. Miller et al. (2020) recorded that the holotype of this species was collected on a small gravel road, probably being drawn out by heavy rainfall prior to collection. Similarly, the specimen from China was also collected on a road during a rainy day. Therefore, we speculate that this species may be easily discovered on rainy days. However, other ecological information about this species is still unknown. It is expected that more specimens of this species will be found in the future to fully understand its natural history and conservation status.

## Supplementary Material

XML Treatment for Achalinus zugorum

## Figures and Tables

**Table 1.** Sequences (COI) used in the phylogenetic analysis of this study.

**Species**	**Locality**	**Voucher**	**Accession**
*A. ater*	Huaping, Guangxi, China	SYSr00852	MN380334
*A. dabieshanensis*	Yuexi, Anhui, China	QHU2024013	PQ507900
*A. damingensis*	Nanning, Guangxi, China	ANU20220009	OP644487
*A. dehuaensis*	Dehua, Fujian, China	YBU13013	MZ442642
*A. emilyae*	Hoanh Bo, Quang Ninh, Vietnam	IEBR4465	MK330857
*A. formosanus*	Taiwan, China	RN2002	KU529452
*A. huangjietangi*	Huangshan, Anhui, China	HSR18030	MT380191
*A. hunanensis*	Huaihua, Hunan, China	CIB119039	OQ848425
*A. jinggangensis*	Fujian, China	QHU 2023011	PQ507882
*A. juliani*	Ha Lang, Cao Bang, Vietnam	IEBRA.2018.8	MK330854
*A. meiguensis*	Mianyang, Sichuan, China	GP835	MZ442641
*A. nanshanensis*	Huaihua, Hunan, China	HNNU230901	OR523368
*A. niger*	Taiwan, China	RN0667	KU529433
*A. ningshanensis*	Ningshan, Shaanxi, China	QHU2023006	PQ507879
*A. panzhihuaensis*	Yanbian, Sichuan, China	KIZ040189	MW664862
*A. pingbianensis*	Honghe, Yunnan, China	YBU18273	MT365521
*A. quangi*	Phu Yen, Son La, Vietnam	ZVNU.2022.08	OQ197471
*A. rufescens*	Hongkong, China	SYSr001866	MN380339
*A. sheni*	Lianyuan, Hunan, China	ANU20230013	OR178146
*A. spinalis*	Hubei, China	QHU 2024023	PQ507894
*A. timi*	Thuan Chau, Son La, Vietnam	IEBRA.2018.10	MK330856
*A. tranganensis*	Ninh Binh, Vietnam	VNUFR.2018.21	MW023086
*A. vanhoensis*	Van Ho, Son La, Vietnam	VNUFR.2019.13	ON677935
*A. yangdatongi*	Wenshan, Yunnan, China	KIZ034327	MW664865
*A. yunkaiensis*	Maoming, Guangdong, China	SYSr001443	MN380329
*A. zugorum*	Bac Me, Ha Giang, Vietnam	IEBR4698	MT502775
*A. zugorum*	Wenshan, Yunnan, China	KIZ2025021	PX130297
*Fimbrios klossi*	Quang Ngai, Vietnam	IEBR3275	KP410744
*Parafmbrios lao*	Louangphabang, Laos	MNHN2013.1002	KP410746
*Xenodermus javanicus*	Sumatera Barat, Indonesia	—	KP410747

**Table 2.** Measurements (in mm) and scale counts of the specimen of *Achalinus zugorum* from China (for abbreviations, see Material and Methods).

**KIZ2025021**		**KIZ2025021**	
SVL	268	FrontalW	2.6
TailL	63	ParietalSL	4.3
TotalL	331	LorealL	1.9
HeadL	9.1	NasalAH	1.2
HeadW	5.3	NasalPH	1.1
SnL	3.4	SL	6/6
SnW	2.3	IL	6/6
EyeD	1.1	ATem	2/2
NarEye	2.2	PTem	2/2
InterorbD	3.5	DS	25-23-23
InternasalSL	1.7	VS	165+5
PrefrontalSL	0.8	SC	61
FrontalL	2.2	Prec	undivided

**Table 3.** Uncorrected pairwise genetic distances (%) calculated from COI sequences.

	**1**	**2**	**3**	**4**	**5**	**6**	**7**	**8**	**9**	**10**	**11**	**12**	**13**
1 *A. ater*													
2 *A. dabieshanensis*	14.8												
3 *A. damingensis*	8.2	15.9											
4 *A. dehuaensis*	16.5	17.4	16.0										
5 *A. emilyae*	11.7	16.6	13.0	15.7									
6 *A. formosanus*	14.1	18.1	14.9	15.7	14.6								
7 *A. huangjietangi*	15.0	8.8	16.3	16.8	14.8	16.2							
8 *A. hunanensis*	7.3	16.8	6.1	14.9	13.2	13.7	16.8						
9 *A. jinggangensis*	12.2	13.8	12.2	14.2	12.7	11.8	11.0	12.3					
10 *A. juliani*	7.1	15.6	8.5	15.2	11.5	13.4	14.8	8.8	12.1				
11 *A. meiguensis*	15.4	17.9	16.8	18.1	15.4	15.6	15.2	16.4	12.9	16.8			
12 *A. nanshanensis*	6.9	15.9	5.8	14.3	13.0	14.4	16.6	5.0	12.2	7.7	17.7		
13 *A. niger*	13.5	14.7	14.3	15.7	12.9	9.0	14.6	13.2	10.6	12.9	13.9	12.8	
14 *A. ningshanensis*	13.0	13.7	12.7	13.2	12.5	12.1	11.6	13.0	8.0	12.4	15.0	13.0	9.5
15 *A. panzhihuaensis*	16.2	16.8	15.5	15.3	16.6	16.0	15.2	16.2	15.5	15.5	11.6	15.1	14.4
16 *A. pingbianensis*	11.8	15.0	11.3	14.8	13.0	14.5	13.0	11.1	11.2	12.2	16.8	11.8	11.7
17 *A. quangi*	11.7	16.9	13.1	15.2	4.0	14.4	15.1	13.2	12.7	12.2	15.2	13.1	12.2
18 *A. rufescens*	12.7	15.4	13.8	14.3	8.0	14.1	14.3	12.1	11.9	12.3	17.3	11.9	12.7
19 *A. sheni*	12.8	14.5	13.8	13.7	13.0	13.2	13.5	12.1	9.0	13.5	14.1	13.6	12.7
20 *A. spinalis*	14.3	15.0	14.0	14.2	13.7	13.5	12.7	13.7	10.6	13.7	16.4	13.6	12.6
21 *A. timi*	13.3	15.8	13.5	15.8	12.9	14.0	14.8	12.0	13.3	13.7	15.8	13.8	12.0
22 *A. tranganensis*	12.7	14.6	13.9	14.2	10.6	17.3	13.7	14.0	13.8	12.3	16.4	13.0	14.9
23 *A. vanhoensis*	13.1	15.7	12.6	15.8	12.3	14.1	14.8	11.5	12.2	13.5	15.6	12.8	12.6
24 *A. yangdatongi*	6.2	16.8	5.6	14.0	12.8	14.4	14.6	5.1	11.7	7.3	17.1	4.4	13.7
25 *A. yunkaiensis*	12.8	14.1	12.5	14.7	13.1	12.3	12.5	12.0	8.6	12.5	15.8	12.5	12.2
26 *A. zugorum* (Holotype)	13.1	14.2	12.8	14.3	12.9	13.7	14.4	11.8	11.6	13.5	15.0	13.0	13.4
27 *A. zugorum* (KIZ2025021)	13.0	13.3	12.5	13.5	12.8	13.1	14.1	11.4	11.1	12.7	15.2	13.0	12.9
	**14**	**15**	**16**	**17**	**18**	**19**	**20**	**21**	**22**	**23**	**24**	**25**	**26**
15 *A. panzhihuaensis*	14.2												
16 *A. pingbianensis*	10.6	14.9											
17 *A. quangi*	11.9	16.9	13.8										
18 *A. rufescens*	12.8	16.0	12.9	7.9									
19 *A. sheni*	9.5	14.6	11.5	13.8	13.1								
20 *A. spinalis*	8.4	14.9	12.5	13.2	12.5	11.4							
21 *A. timi*	11.9	15.5	12.2	13.1	13.9	13.8	13.7						
22 *A. tranganensis*	12.9	16.4	13.3	11.9	11.5	13.0	14.5	13.5					
23 *A. vanhoensis*	12.1	15.5	10.8	12.3	13.8	14.1	12.4	5.0	13.3				
24 *A. yangdatongi*	12.0	15.5	11.3	12.6	11.5	13.7	13.5	13.1	12.8	11.3			
25 *A. yunkaiensis*	9.4	15.7	11.6	13.6	13.3	6.3	11.2	14.1	13.5	13.6	12.0		
26 *A. zugorum* (Holotype)	11.3	15.3	10.9	12.6	13.5	10.9	12.9	13.4	12.5	12.0	12.2	10.9	
27 *A. zugorum* (KIZ2025021)	11.3	14.6	10.4	12.4	13.1	10.9	12.9	13.4	12.1	12.0	12.2	11.2	2.9
